# Extensively Drug-Resistant Tuberculosis with Conflicting Resistance Testing Results, Lesotho

**DOI:** 10.3201/eid3111.250885

**Published:** 2025-11

**Authors:** Kwonjune J. Seung, Meseret Asfaw, Mikanda Kunda, Llang Bridget Maama-Maime, Joalane Makaka, Mabatloung Mofolo, Stephane Mpinda, Melino Ndayizigiye, Shaheed Vally Omar, Prithiv Prasad, Praharshinie Rupasinghe, Chase Yarbrough, Lawrence Oyewusi

**Affiliations:** Brigham and Women’s Hospital, Boston, Massachusetts, USA (K. Seung, C. Yarbrough); Partners In Health, Maseru, Lesotho (M. Asfaw, M. Kunda, J. Makaka, M. Mofolo, S. Mpinda, M. Ndayizigiye, P. Prasad); Government of Lesotho Ministry of Health and Social Welfare, Maseru (L.B. Maama-Maime, L. Oyewusi); National Institute for Communicable Diseases, Johannesburg, South Africa (S.V. Omar); Institute of Tropical Medicine, Antwerp, Belgium (P. Rupasinghe)

**Keywords:** tuberculosis, extensively drug-resistant tuberculosis, bacteria, tuberculosis and other mycobacteria, antimicrobial resistance, genotypic resistance testing, phenotypic resistance testing, Lesotho

## Abstract

A patient with extensively drug-resistant tuberculosis in Lesotho recovered successfully after failed treatment with bedaquiline, delamanid, linezolid, and clofazimine. Whole-genome sequencing and broth microdilution testing results were not in agreement, illustrating the urgent need for studies that correlate phenotypic and genotypic resistance testing with clinical response.

New drugs and regimens for treating tuberculosis (TB) have transformed the way in which healthcare providers manage multidrug-resistant (MDR) TB. Bedaquiline, delamanid, and pretomanid are among the newest drugs developed specifically for treating TB. Other drugs, like linezolid and clofazimine, have demonstrated activity against *Mycobacterium tuberculosis* and have therefore gained status as potential treatments for the disease. Healthcare professionals have reported excellent treatment outcomes in patients receiving those drugs, even in low-resource settings that have the highest burden of MDR TB (*1*,*2*). Resistance to those drugs, however, has been increasing faster than access to accurate laboratory resistance testing. Bedaquiline resistance is increasingly common and problematic, given the drug’s prominence in most treatment regimens for MDR TB. (*3*).

In 2016, physicians referred a man in his late 30s to the Botsabelo MDR TB referral hospital in Maseru, Lesotho, for suspicion of drug-resistant TB. The man’s only previous exacerbation of TB was 2 years earlier, when he received a standard treatment regimen of 4 first-line drugs. At that time, chest radiographs showed bilateral upper lobe infiltrates, more extensive on the right side; PCR testing with Xpert MTB/RIF assay (Cepheid, https://www.cepheid.com) showed resistance to rifampin. He had HIV infection (CD4 115 cells/µL, viral load 20,000 copies/mL), which appeared to be poorly managed because of inconsistent adherence to abacavir, lamivudine, and efavirenz, especially during episodes of binge drinking.

When the patient sought care in 2016, we performed qualitative in vitro testing with GenoType MTBDR*sl* (Hain Lifescience, https://www.hain-lifescience.de), which showed no evidence of mutations conferring resistance to fluoroquinolones or injectables, so we started a standard treatment regimen for MDR TB: pyrazinamide, kanamycin, levofloxacin, prothionamide, cycloserine, and para-aminosalicylic acid (Figure, panel A). Clinical and bacteriologic response was poor. Repeat testing with GenoType MTBDR*sl* showed resistance to fluoroquinolones but not to injectables; we adjusted the drug regimen at month 10 to include bedaquiline and linezolid. At month 14, we added delamanid and clofazimine. Nevertheless, sputum cultures were persistently TB positive. At month 19, BACTEC MGIT (BD, https://www.bd.com) analysis of a sputum isolate sent to the South Africa National Institute for Communicable Diseases (NICD; Johannesburg, South Africa) revealed susceptibility to all second-line TB drugs except moxifloxacin (0.5 μg/mL).

**Figure:**
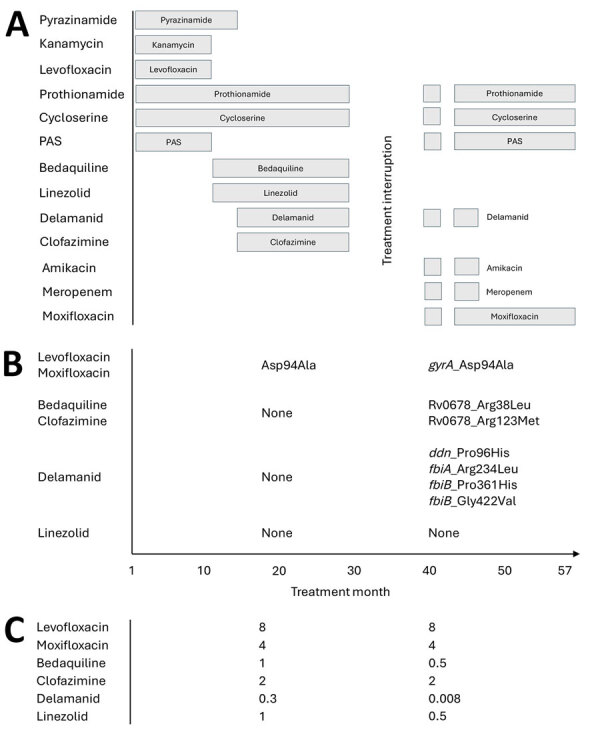
Data from patient with extensively drug-resistant tuberculosis with conflicting resistance testing results, Lesotho. A) Treatment regimen of the patient. B–C) Drug-resistance mutations (B) and MICs (C) of *Mycobacterium tuberculosis* isolates collected from the patient during months 19 and 42 of treatment. Whole-genome sequence analysis for drug resistance was conducted at the South Africa National Institute for Communicable Diseases (Johannesburg, South Africa), and MIC testing with broth microdilution was conducted at the Institute of Tropical Medicine (Antwerp, Belgium).

After month 29 of follow-up, the patient stopped attending the program, but he returned 8 months later, when his clinical condition worsened. Chest radiographs showed a large, right-sided middle lobe cavity, bilateral lymphadenopathy, and bilateral infiltrates. We consulted thoracic surgeons in Durban, South Africa, about resective surgery, but a right pneumonectomy was not an option because the left lung was also compromised.

In month 42, a sputum isolate sent to NICD confirmed resistance (BACTEC MGIT) to additional second-line drugs, including levofloxacin, moxifloxacin (0.5 μg/mL), bedaquiline, linezolid, and clofazimine. The isolate analysis revealed the TB strain to be susceptible to moxifloxacin at 1.0 μg/mL; delamanid was not tested. We subsequently changed the patient’s drug regimen to prothionamide, cycloserine, para-aminosalicylic acid, delamanid, amikacin, meropenem, and high-dose moxifloxacin (800 mg/d). We administered meropenem through a peripheral intravenous tube that was changed every few weeks. The patient improved clinically and bacteriologically, and he completed treatment in month 57, after 5 consecutive negative sputum cultures.

After the patient’s recovery, we sent sputum isolates from month 19 and month 42 to NICD, where technicians performed whole-genome sequencing (WGS) analysis (Figure, panel B). We also sent subcultures to the Institute of Tropical Medicine (Antwerp, Belgium) for MIC testing with broth microdilution (Figure, panel C). WGS revealed no resistance mutations for any of these drugs in the month 19 sputum sample, but the MICs of bedaquiline, clofazimine, and delamanid were above the breakpoints typically considered to be resistant (*4*). In the month 42 sputum sample, WGS found resistance mutations to bedaquiline and clofazimine, consistent with the MIC testing, but there were multiple resistance mutations to delamanid, even though the MIC of delamanid was below the typical breakpoint for resistance. WGS and MIC testing were consistent for fluoroquinolones (resistant) and linezolid (susceptible) in sputum samples from both months.

Complicating interpretation of WGS is the fact that many resistance mutations for the new and repurposed drugs have not yet been discovered. In the patient we treated, who was symptomatic and bacteriologically sputum positive for many months on a regimen containing bedaquiline, clofazimine, delamanid, and linezolid, the mutation conferring resistance to fluoroquinolones, Asp94Ala, is well known, but none of the other mutations found (Figure, panel C) have been previously reported in the scientific literature as conferring resistance to bedaquiline/clofazimine (Rv0678_Arg38Leu, Rv0678_Arg123Met) or delamanid (ddn_Pro96His, fbiA_Arg234Leu, fbiB_Pro361His, fbiB_Gly422Val) (*5*). We considered those to be true resistance mutations because they are located in relevant genes, and the clinical, bacteriologic, and radiologic evidence is consistent with resistance acquisition.

Even with new drugs and regimens, treating MDR TB will continue to be challenging. As both phenotypic and genotypic resistance testing for new and repurposed TB drugs continues to evolve, so must our understanding of how resistance testing correlates with clinical response.
